# Potential of Machine Learning for Predicting Sleep Disorders: A Comprehensive Analysis of Regression and Classification Models

**DOI:** 10.3390/diagnostics14010027

**Published:** 2023-12-22

**Authors:** Raed Alazaidah, Ghassan Samara, Mohammad Aljaidi, Mais Haj Qasem, Ayoub Alsarhan, Mohammed Alshammari

**Affiliations:** 1 Department of Data Science and AI, Faculty of Information Technology, Zarqa University, Zarqa 13110, Jordan; razaidah@zu.edu.jo (R.A.); m.hajqasem@zu.edu.jo (M.H.Q.); 2 Department of Computer Science, Faculty of Information Technology, Zarqa University, Zarqa 13110, Jordan; mjaidi@zu.edu.jo; 3 Department of Information Technology, Faculty of Prince Al-Hussein Bin Abdullah II for Information Technology, The Hashemite University, Zarqa 13133, Jordan; ayoubm@hu.edu.jo; 4 Faculty of Computing and Information Technology, Northern Border University, Rafha 91431, Saudi Arabia

**Keywords:** classification, learning strategies, machine learning, sleep disorders, regression

## Abstract

Sleep disorder is a disease that can be categorized as both an emotional and physical problem. It imposes several difficulties and problems, such as distress during the day, sleep-wake disorders, anxiety, and several other problems. Hence, the main objective of this research was to utilize the strong capabilities of machine learning in the prediction of sleep disorders. In specific, this research aimed to meet three main objectives. These objectives were to identify the best regression model, the best classification model, and the best learning strategy that highly suited sleep disorder datasets. Considering two related datasets and several evaluation metrics that were related to the tasks of regression and classification, the results revealed the superiority of the MultilayerPerceptron, SMOreg, and KStar regression models compared with the other twenty three regression models. Furthermore, IBK, RandomForest, and RandomizableFilteredClassifier showed superior performance compared with other classification models that belonged to several learning strategies. Finally, the Function learning strategy showed the best predictive performance among the six considered strategies in both datasets and with respect to the most evaluation metrics.

## 1. Introduction

Sleep is an important natural activity for humans and plays a very important role in everybody’s health [1]. Our body supports healthy brain functionality and maintains the necessary physical health while sleeping [2]. Moreover, sleeping is very important for body development and growth, especially for children and teenagers. Sleeping really impacts the way of thinking, working, learning, reacting, and many other aspects of daily life. It also affects the circulation, immunity, and respiratory systems of our bodies [3].

On the other hand, lack of sleep (sleep disorder) causes several problems and difficulties in daily life [4]. To name a few, sleep disorders increase the levels of hormones that control hunger, increase consumption of sweet, salty, and fatty foods, decrease the levels of physical activity, and increase the risk of obesity, stroke, and heart disease [5]. It may also cause stress, fatigue, and functional weaknesses [6,7]. Moreover, sleep disorder is one of the main reasons for sleep apnea. According to recent statistics from U.S. census data, more than 140 million (70 million men, 50 million women, and 20 million children) snore mostly because of sleep apnea. Globally, around 936 million adults suffer from mild to severe sleep apnea. Moreover, according to several global research works, around 10%, even up to 30% of the world’s population suffer from sleep disorder, and in some countries the percentage may reach 60%. Furthermore, sleep disorder is nearly 7% higher among women than among men. Finally, sleep disorder represents a global epidemic that threatens the quality of life and health for around 45% of the world’s population.

Based on the recent literature of sleep disorder, it can be noted that the following research dominates this field. Firstly, the relationship between COVID-19 and sleep disorder. Secondly, searching for new tests other than obstructive sleep apnea (OSA) that is less costly and more comfortable to possible patients is an urgent need. Finally, the utilization of machine learning and wearable devices with fewer sensors for sleep disorder diagnosis at home without the need to sleep in specific sleep centers.

Consequently, this research aimed to provide additional knowledge and contribute to the solution of the sleep disorder problem through utilizing machine learning capabilities in the prediction task of sleep disorders [8]. In specific, this research was interested in three main objectives:

1. To identify the best regression model that highly suits disorder datasets among twenty three different regression models
2. To identify the best classification model that highly suits disorder datasets among twenty nine different classification models
3. To identify the best learning strategy that highly suits disorder datasets among six different well-known strategies.

Therefore, this research considered two main machine learning tasks: regression and classification. Both tasks were used to predict unknown values [9]. The difference was that regression was used to predict numeric values, while classification was used to predict non-numeric values [10,11].

Regarding the classification task, it was defined as the ability to predict the class label for unseen cases or examples accurately [12,13]. Classification was of two types: single label classification (SLC) and multi label classification (MLC). The former type associates every instance or case with only one class label, while the latter may associate an instance or example with more than one class label [14,15,16].

SLC was also divided into two subtypes: binary classification and multiclass classification [17]. For binary classification, the total number of class labels in the dataset was only two [18,19]. For multiclass classification, the number of class labels in the dataset was more than two. The dataset in this research belonged to the multiclass classification [20].

Regarding the regression task, it was defined as the task of understanding the relationship between the objective variable (the dependent variable) and the considered variables and features in the dataset (independent variables) [21]. The objective variable in regression must be continuous; it was a main supervised task in machine learning that aimed to predict the value of a continuous variable based on a set of known variables [22]. Regression has many real life applications, such as forecasting house prices [23], predicting users’ trends [24], and predicting interest rates [25,26], among several other others.

To achieve the first objective, this research considered twenty three regression models that belonged to four learning strategies. These regression models were evaluated and compared using two datasets with respect to five well-known evaluation metrics. To achieve the second objective, twenty nine classification models were evaluated and compared with respect to five popular metrics in the domain of classification.

The rest of the paper is organized as follows: Section 2 reviews the most recent work related to sleep disorder detection using machine learning techniques. Section 3 describes the research methodology and the considered datasets, and it provides the empirical results, followed by the main findings. Section 4 concludes and suggests a future direction.

## 2. Related Work

Everyone requires sleep. It is a crucial component of how our bodies work. You may require more or less sleep than others, but doctors advise people to get seven to nine hours per night. Most people face a problem with sleeping called a sleep disorder. Sleep disorders are situations in which the usual sleep pattern or sleep behaviors are disrupted, and the main sleep disorders include insomnia, hypersomnia, obstructive sleep apnea, and parasomnias.

In addition to contributing to other medical concerns, several of these disorders may also be signs of underlying mental health problems, which led researchers to do a lot of research. In [27], the authors presented a thorough study of the relationship between vitamin D and sleep problems in children and adolescents who suffer from sleep disorders such as insomnia, obstructive sleep apnea (OSA), restless leg syndrome (RLS), and other sleep disorders. The research synthesized information regarding the role and mechanism of the action of vitamin D. A review of the use of melatonin and potential processes in the sleep disturbances of Parkinson’s disease patients can be found in [28].

In [29], researchers conducted a systematic study and meta-analysis to identify the key elements contributing to sleep and anxiety problems during the COVID-19 pandemic lockdown. Additionally, the study aimed to forecast potential correlations and determinants in conjunction with results connected to COVID-19 pandemic-induced stress and difficulties and analyzed the various symptoms and complaints that people experienced with regard to their sleep patterns. The Pittsburgh Sleep Quality Index (PSQI), machine learning algorithms, and the general assessment of anxiety disorders were used to analyze the outcomes. The study looked at a significant correlation between symptoms such as poor sleep, anxiety, depressive symptoms, and insomnia, as well as the COVID-19 pandemic lockdown.

In [30], a cross-validated model was proposed for classifying sleep quality based on the goal of the act graph data. The final classification model demonstrated acceptable performance metrics and accuracy when it was assessed using two machine learning techniques: support vector machines (SVM) and K-nearest neighbors (KNN). The findings of this research can be utilized to cure sleep disorders, create and construct new methods to gauge and monitor the quality of one’s sleep, and enhance current technological devices and sensors.

In [31], they proposed a general-purpose sleep monitoring system that may be used to monitor bed exits, assess the danger of developing pressure ulcers, and monitor the impact of medicines on sleep disorders. Additionally, they contrasted a number of supervised learning algorithms to find which was most appropriate in this situation. The experimental findings from comparing the chosen supervised algorithms demonstrated that they can properly infer sleep duration, sleep postures, and routines with a fully unobtrusive method.

In [32], they proposed a reliable approach for classifying different stages of sleep using a sleep standard called AASM based on a single channel of electroencephalogram (EEG) data. The use of statistical features to analyze the sleep characteristics and the three distinct feature combinations utilized to categorize the two-state sleep phases were the main contributions of this work. Both patients with sleep disorders and healthy control subjects participated in three separate trials with three distinct sets of characteristics. As a result, many machine learning classifiers were developed to categorize the various stages of sleep.

## 3. Materials and Methods

This section represents the core of this research. Firstly, the datasets are described along with the required preprocessing steps. Then, the evaluation results for the twenty three considered regression models are provided and discussed. After that, a comparative analysis among twenty nine classification models (classifiers) was conducted and analyzed. Finally, a discussion regarding the most interesting findings is carried out.

**Figure 1:**
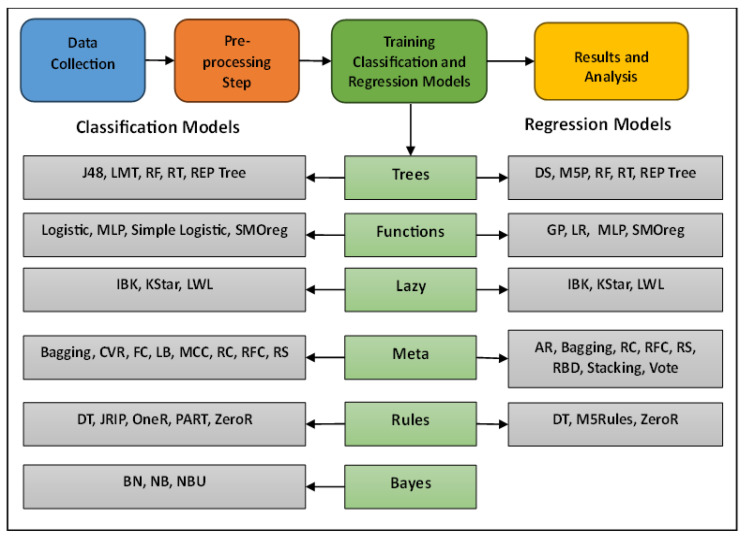
Main phases in research methodology.

Regarding the experimental design, all classification and regression models were used with their default settings and parameters except for the IBK algorithm, where the KNN parameter was changed from 1 to 3. Moreover, the considered models were implemented using the Python programming language. Experiments have also been conducted on the Intel i3 core. Finally, to handle the problem of missing values, all missing values were estimated to be the average of the values within the same class. The main phases of research methodology are shown in Figure 1.

### Datasets and Preprocessing Step

Two datasets were considered in this research. The first one (Dataset 1) consists of 62 cases and 11 features. This dataset was an extended version of the second dataset (Dataset 2), where three features were added and considered. Both datasets suffer from missing values. The main goal of collecting the datasets was to study sleeping patterns in mammals. Another main goal behind collecting this data was to identify the main factors affecting the quality of sleep and to diagnose the main risks regarding sleep disorders. The main features (attributes) in both datasets were: body weight, brain weight, predation index, sleep exposure index, gestation time, and danger index. All of these features were numerical and both datasets consisted of five class labels. Both datasets are graciously shared on Kaggle and freely available at the following URL: (https://www.kaggle.com/datasets/volkandl/sleep-in-mammals, accessed on 12 December 2023). Table 1 summarizes the main characteristics of the considered datasets, while Table 2 provides more information regarding the features in both datasets.

Originally, both datasets were of type regression. Nevertheless, a mapping was carried out to convert the objective feature from being a number to a class variable (string). For example, instead of having ‘1’ as a value for the ‘overall danger index’ feature, it was converted to ‘A’, and instead of having ‘5’ as a value for the ‘overall danger index’ feature, it was converted to ‘E’.

**Figure 2:**
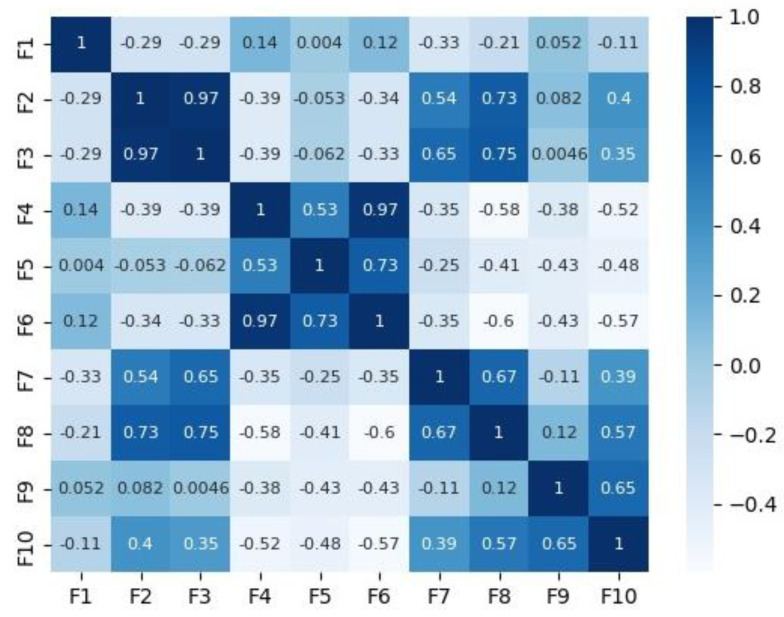
Correlation matrix for Dataset 1.

**Figure 3:**
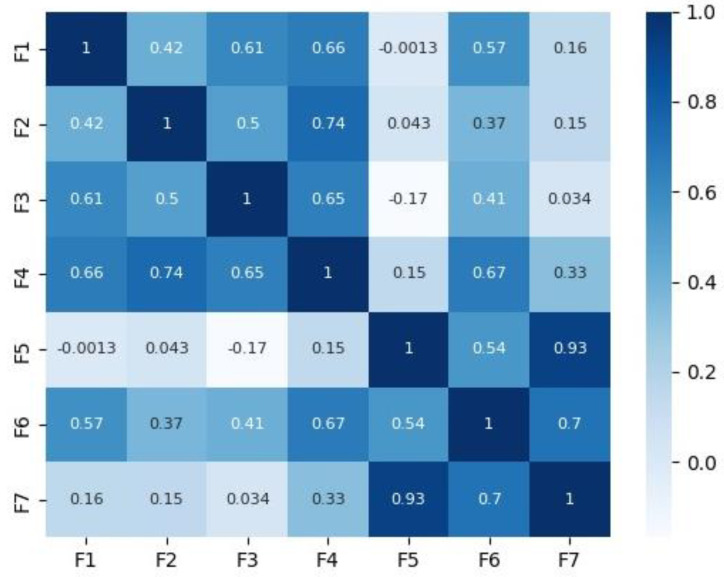
Correlation matrix for Dataset 2.

Figure 2 and Figure 3 depict the correlation matrices for Dataset 1 that consisted of 10 features (excluding the class feature), and Dataset 2 that consisted of 7 features (excluding the class feature) respectively.

## 4. Results

### 4.1. Identifying the Best Regression Model

Identifying the best regression model was the main objective of this research. To meet this objective, twenty three regression models were considered and evaluated. These models belonged to five well-known strategies.

The Function learning strategies were represented through four models: Gaussian processes, linear regression, multilayer perception, and SMOreg. Three models were used to represent the Lazy learning strategy: IBK, KStar, and LWL. For the meta-learning strategy, the following eight regression models were considered: AdditiveRegression, Bagging, RandomCommittee, RandomizableFilteredClassifier, RandomSubSpace, RegressionByDiscretization, Stacking, and Vote. The Rules learning strategy was represented using the following models: DecisionTable, M5Rules, and ZeroR. Finally, five models were used to represent Tree learning strategies (DecisionStump, M5P, RandomForest, RandomTree, and REPTree).

It is worth mentioning that all these models were used with their default settings and parameters, except for the IBK algorithm, where the KNN parameter was changed from 1 to 3.

The evaluation phase of the considered regression models was carried out on both datasets (Dataset 1 and Dataset 2) with respect to five different and well-known evaluation metrics such as correlation coefficient (CC), mean absolute error (MAR), root mean squared error (RMSE), relative absolute error (RAE), and root relative squared error (RRSE). These metrics were computed using the following equations:

(1) $$CC=\frac{\sum \left(x_{i}-\bar{x})(y_{i}-\bar{y}\right)}{\sqrt{\sum {\left(x_{i}-\bar{x}\right)}^{2}}\sum {\left(y_{i}-\bar{y}\right)}^{2}}$$

(2) $$MAE=\frac{\sum_{i=1}^{n}\left|y_{i\;}-x_{i}\right|}{n}\;$$

(3) $$RMSE=\sqrt{\frac{\sum_{i=1}^{n}{\left\|y\left(i\right)-\hat{y}\;(i)\right\|}^{2}}{N}}$$

RAE = mean of the absolute value of the actual forecast errors/mean of the absolute values of the naive model’s forecast errors(4)

(5) $$RPSE=\sqrt{\frac{\sum_{i=1}^{n}{\left(y_{i\;}-{\hat{y}}_{i}\right)}^{2}}{\sum_{i=1}^{n}{\left(y_{i\;}-{\bar{y}}_{i}\right)}^{2}}}\;$$

Table 3 depicts the evaluation results for CC metrics in both datasets using twenty three regression models.

According to Table 1 and considering Dataset 1, several models achieved strong results, such as GaussianProcesses, MultilayerPerceptron, SMOreg, IBK, RegressionByDiscretization, and RandomForest. The best regression model, according to the table, was the MultilayerPerceptron regression model, which belonged to the Function learning strategy. Moreover, the second best model belonged to the Function strategy, which was SMOreg. For Dataset 2, both MultilayerPerceptron and SMOreg achieved the best results among the twenty three considered regression models.

Table 4 represents the MAE results for the twenty three regression models in both datasets. According to Table 4 and considering Dataset 1, RegressionByDiscretization which belonged to the meta-learning strategy, achieved the best (lowest) results compared with the other twenty two regression models. MultilayerPerceptron achieved the second best value. It is worth mentioning that MAE itself was not sufficient to assess the regression models. Therefore, this research considered other evaluation metrics. For dataset 2, SMOreg achieved the best results, followed by the KStar algorithm. Both models belonged to Lazy learning strategy.

Table 5 shows the results for the RMSE metric in both datasets using the same twenty three regression models. For the RMSE metric, the lower the value, the better the performance. From Table 5, and considering Dataset 1, MultilayerPerceptron and SMOreg from the Function learning strategy achieved the best two results, respectively. Moreover, RegressionByDiscretization, GaussianProcesses, and IBK achieved acceptable results compared with the other regression models considered in this research. For Dataset 2, the IBK and KStar models achieved the best two results, respectively.

Table 6 depicts the empirical results for the RAE metric, which considered twenty three regression models and two datasets. For the RAE metric, the lower the value, the better the predictive performance. According to Table 6, and considering Dataset 1, MultilayerPerceptron and SMOreg achieved the best two results, respectively. Both regression models belonged to the Function learning strategy. The third regression model was IBK, which belonged to the Lazy learning strategy. For Dataset 2, SMOreg achieved the best RAE result, followed by the KStar model.

Table 7 represents the RRSE evaluation results for the twenty three considered regression models in both datasets. For this metric, the lower the value, the better the predictive performance. Considering Dataset 1, and according to Table 7, MultilayerPerceptron and SMOreg were the best two regression models, respectively. RegressionByDiscretization regression model from the meta-learning strategy achieved the third best results on dataset 1.

Considering Dataset 2, KStar from the Lazy learning strategy achieved the best RRSE result, followed by SMOreg from the Function learning strategy.

Table 8 summarizes the previous tables in order to identify the best regression model among the twenty three considered models. For Table 8, MLP is short for MultilayerPerceptron and RBD is short for RegressionByDiscretization.

According to Table 8, the MLP model achieved the best results on Dataset 1, while SMOreg achieved the second best results on the same dataset. For dataset 2, SMOreg achieved the best results, followed by the KStar model. Hence, it can be concluded that ensemble learning was the best way to handle the prediction task for sleeping disorder datasets with respect to utilizing the following models: MLP, SMOreg, and KStar.

### 4.2. Identifying the Best Classification Model

This section aimed to identify the best classification algorithm to use with the problem of sleep disorders. The evaluation phase in this section considered twenty nine classification models that belonged to six learning strategies.

These classification models were: BayesNet, NaiveBayes, NaiveBayesUpdateable from Bayes learning strategy. Logistic, MultilayerPerceptron, SimpleLogistic, and SMO from Functions learning strategy. IBK, KStar, and LWL from the Lazy learning strategy. Bagging, ClassificationViaRegression, FilteredClassifier, LogitBoost, MultiClassClassifier, RandomCommittee, RandomizableFilteredClassifier, RandomSubSpace, and Vote from Meta Learning Strategy. DecisionTable, JRip, OneR, PART, and ZeroR were from the Rules learning strategy. J48, LMT, RandomTree, RandomForest, and REPTree from the Trees learning strategy

Moreover, the evaluation phase for this section considered five different and well-known metrics. These metrics were accuracy, precision, recall, F1-measure, and Matthew’s correlation coefficient (MCC). The considered evaluation metrics were computed using the following equations:

(6) $$Accuracy=\frac{TP+TN}{TP+TN+FP+FN}$$

(7) $$Precision=\frac{TP}{TP+FP}$$

(8) $$Recall=\frac{TP}{TP+FN}$$

F1 = Measure = (2 × Precision × Recall)/(Precision + Recall)(9)

MCC = (*TP* × *TN* − *FP* × *FN*)/√(*TP* + *FP*) (*TP* + *FN*)(*TN* + *FP*)(*TN* + *FN*) (10)

For all the previously mentioned metrics, the higher the value, the better the performance of the classification model.

Table 9 shows the accuracy and precision results for the twenty nine considered classification models on the two considered datasets. According to Table 9, IBK and RandomForest classifiers achieved the highest accuracy and precision results on dataset 1. For Dataset 2, IBK showed the best results among the twenty nine considered classifiers with respect to accuracy and precision metrics. Moreover, RandomizableFilteredClassifier showed the best accuracy result on Dataset 2 and the second best precision result on the same dataset.

**Figure 4:**
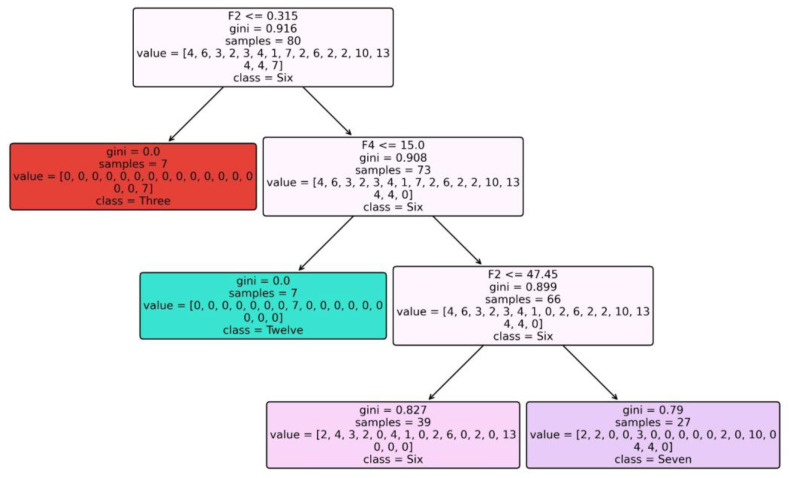
Tree constructed for Dataset 1 when using RandomTree classifier.

Figure 4 depicts the constructed Tree for Dataset 1 when using RandomTree as a classification model.

Table 10 depicts the evaluation results for the twenty nine considered classifiers in both datasets, considering recall and F1-measure metrics. According to Table 10, the IBK classifier achieved the best recall results in both datasets and the best F1-measure result on Dataset 1. RandomForest classifier achieved the best F1-measure result on Dataset 2 in addition to the best recall result on Dataset 1 along with the IBK classifier.

Table 11 depicts the MCC results for the considered classifiers in both datasets. Based on Table 11, the IBK classifier that belonged to the Lazy learning strategy achieved the best MCC results on both considered datasets. Moreover, the RandomForest classifier, which belonged to the Trees learning strategy, achieved the best MCC result on Dataset 2.

Table 12 summarizes the best results obtained in Table 9 and Table 11 with respect to the five evaluation metrics considered in both datasets. For Table 12, RF stands for Random Forest classifier, and RFC stands for RandomizableFilteredClassifier.

According to Table 12, IBK and RandomForest classifiers were the best classification models to handle dataset 1, respectively, while IBK, RandomizableFilteredClassifier, and RandomForest were the best classification models to handle dataset 2.

## 5. Discussion

In this section, a comparative analysis regarding the best regression and classification models that could handle the task of predicting the problem of sleep disorders was introduced. The analysis considered two datasets with respect to several evaluation metrics.

Regarding the best regression model to use, it was clearly noted that no single regression model showed a general high performance considering all the metrics in both datasets. Therefore, it is highly recommended to utilize ensemble methods for this task with consideration for the best regression models, as shown in Section 4.1 (Multilayer Perceptron, SMOreg, and KStar).

For the best classification model to use, the IBK classification model showed superior performance compared with the other models. Nevertheless, other classification models showed excellent performance, such as RandomForest and RandomizableFilteredClassifier. Hence, it is highly recommended to utilize these three classification models (IBK, RandomForest, and RandomizableFilteredClassifier) in ensemble learning for handling the problem of classifying disordered sleep.

Moreover, regarding the best learning strategy to use with the problem of sleep disorder, the following strategies showed excellent performance: Lazy, Functions, Trees, and Meta. In depth, Table 13 depicts the average results for the considered models with respect to the learning strategies they belong to. The shaded rows represent Dataset 2, while the unshaded rows represent Dataset 1.

According to Table 13, the Lazy learning strategy was the best learning strategy to use with the regression task for disorder datasets, considering the five metrics. Functions was the second best learning strategy.

Considering the classification task, it is clearly seen from Table 13 that the best choice was to consider dataset 1, while the Tree strategy was the best choice when considering dataset 2, and for all five evaluated metrics. The conclusion that could be drawn is that the Function strategy was more suitable for datasets that have a large number of features, while the Trees learning strategy was more efficient for use with datasets that have a smaller number of features.

Once again, based on Table 13, the Function strategy showed superior performance considering the two considered tasks (regression and classification). Therefore, it was the most appropriate strategy to use with the prediction task of disorder datasets.

Finally, it is highly recommended to conduct more integrated research, considering experts from the machine learning domain and the sleeping disorder domain. Considering new features other than the features considered in the utilized datasets is also highly recommended.

## 6. Conclusions and Future Work

Sleep disorders involve problems with the amount, timing, and quality of sleep, which results in several daytime problems such as fatigue, stress, and impairment in functioning. This research aimed to add knowledge to this domain by investigating the applicability of machine learning techniques in the domain of sleep disorders. Mainly, three objectives were considered in this research. These objectives were to identify the best regression model, the best classification model, and the best learning strategy to handle the sleep disorders dataset. The results showed that MultilayerPerceptron, SMOreg, and KStar were the best regression models, and IBK, RandomForest, and RandomizableFilteredClassifier were the best classification models. Finally, the Function learning strategy showed superior performance compared with the other strategies, considering both regression and classification tasks in both datasets, with strong competition from the Lazy and Trees strategies. For future work, an ensemble learning model that consists of the best regression and classification models is highly recommended.

## Figures and Tables

**Table 1:** Datasets characteristics.

Name	Dataset 1	Dataset 2
Type	Classification, Regression	Classification, Regression
Instances	62	62
Features	11	8
Missing Values	Yes	Yes

**Table 2:** Features and main characteristics.

No.	Name	Type	Minimum	Maximum
1	Species	Nominal	-	-
2	Body weight (kg)	Real	0.005	6654
3	Brain weight (g)	Real	0.14	5712
4	Slow wave (h/day)	Real	2.1	17.9
5	Paradoxical (h/day)	Real	0	6.6
6	Total sleep (h/day)	Real	2.6	19.9
7	Maximum life span (years)	Real	2	100
8	Gestation time (days)	Real	12	645
9	Predation index (1–5)	Integer	1	5
10	Exposure index (1–5)	Integer	1	5
11	Overall danger index (1–5)	Integer	1	5

**Table 3:** CC Results using twenty three regression models on both datasets.

Strategy	Model	Dataset 1	Dataset 2
		CC	CC
Functions	GaussianProcesses	0.929	0.605
	LinearRegression	0.570	0.576
	MultilayerPerceptron	0.954	0.699
	SMOreg	0.950	0.684
Lazy	IBK	0.911	0.634
	KStar	0.818	0.679
	LWL	0.811	0.628
Meta	AdditiveRegression	0.845	0.494
	Bagging	−0.249	0.655
	RandomCommittee	0.837	0.639
	RandomizableFilteredClassifier	0.348	0.602
	RandomSubSpace	0.859	0.609
	RegressionByDiscretization	0.933	0.538
	Stacking	−0.287	−0.497
	Vote	−0.287	−0.497
Rules	DecisionTable	0.859	0.527
	M5Rules	0.000	0.587
	ZeroR	−0.287	−0.497
Trees	DecisionStump	0.737	0.485
	M5P	0.000	0.588
	RandomForest	0.903	0.608
	RandomTree	0.759	0.453
	REPTree	−0.287	0.416

**Table 4:** MAE results using twenty three regression models on both datasets.

Strategy	Model	Dataset 1	Dataset 2
		MAE	MAE
Functions	GaussianProcesses	0.445	3.089
	LinearRegression	1.111	2.975
	MultilayerPerceptron	0.328	3.128
	SMOreg	0.351	2.747
Lazy	IBK	0.355	2.847
	KStar	0.596	2.752
	LWL	0.694	2.793
Meta	AdditiveRegression	0.568	3.401
	Bagging	1.290	2.804
	RandomCommittee	0.867	2.923
	RandomizableFilteredClassifier	1.177	3.390
	RandomSubSpace	0.918	2.969
	RegressionByDiscretization	0.293	3.314
	Stacking	1.277	3.741
	Vote	1.277	3.741
Rules	DecisionTable	0.540	3.022
	M5Rules	1.290	2.886
	ZeroR	1.277	3.741
Trees	DecisionStump	0.811	3.306
	M5P	1.290	2.885
	RandomForest	0.816	2.953
	RandomTree	0.730	3.958
	REPTree	1.277	3.415

**Table 5:** Root mean squared error coefficient results using twenty three regression models on both datasets.

Strategy	Model	Dataset 1	Dataset 2
		RMSE	RMSE
Functions	GaussianProcesses	0.561	3.855
	LinearRegression	1.276	4.039
	MultilayerPerceptron	0.435	3.891
	SMOreg	0.451	3.619
Lazy	IBK	0.596	3.335
	KStar	0.834	3.478
	LWL	0.848	3.656
Meta	AdditiveRegression	0.806	4.430
	Bagging	1.455	3.761
	RandomCommittee	1.014	3.562
	RandomizableFilteredClassifier	1.571	4.415
	RandomSubSpace	1.040	3.629
	RegressionByDiscretization	0.530	3.968
	Stacking	1.443	4.696
	Vote	1.443	4.696
Rules	DecisionTable	0.739	3.923
	M5Rules	1.481	3.850
	ZeroR	1.443	4.696
Trees	DecisionStump	0.991	4.076
	M5P	1.481	3.847
	RandomForest	0.949	3.652
	RandomTree	0.940	5.068
	REPTree	1.443	4.299

**Table 6:** Relative absolute error results using twenty three regression models on both datasets.

Strategy	Model	Dataset 1	Dataset 2
		RAE	RAE
Functions	GaussianProcesses	34.838	82.556
	LinearRegression	86.974	79.514
	MultilayerPerceptron	25.648	83.607
	SMOreg	27.455	73.415
Lazy	IBK	27.779	76.082
	KStar	46.653	73.560
	LWL	54.301	74.643
Meta	AdditiveRegression	44.449	90.902
	Bagging	100.948	74.951
	RandomCommittee	67.888	78.120
	RandomizableFilteredClassifier	92.176	90.598
	RandomSubSpace	71.827	79.364
	RegressionByDiscretization	22.921	88.588
	Stacking	100.000	100.000
	Vote	100.000	100.000
Rules	DecisionTable	42.295	80.772
	M5Rules	101.014	77.135
	ZeroR	100.000	100.000
Trees	DecisionStump	63.493	88.351
	M5P	101.014	77.097
	RandomForest	63.900	78.928
	RandomTree	57.124	105.800
	REPTree	100.000	91.268

**Table 7:** Root relative squared error results using twenty three regression models on both datasets.

Strategy	Model	Dataset 1	Dataset 2
		RRSE	RRSE
Functions	GaussianProcesses	38.906	82.097
	LinearRegression	88.471	86.005
	MultilayerPerceptron	30.147	82.860
	SMOreg	31.261	77.065
Lazy	IBK	41.287	84.413
	KStar	57.792	74.074
	LWL	58.755	77.849
Meta	AdditiveRegression	55.858	94.345
	Bagging	100.865	73.712
	RandomCommittee	70.285	78.853
	RandomizableFilteredClassifier	108.880	94.027
	RandomSubSpace	72.113	77.283
	RegressionByDiscretization	36.720	84.498
	Stacking	100.000	100.000
	Vote	100.000	100.000
Rules	DecisionTable	51.193	83.544
	M5Rules	102.653	81.980
	ZeroR	100.000	100.000
Trees	DecisionStump	68.667	86.801
	M5P	102.653	81.929
	RandomForest	65.769	77.767
	RandomTree	65.167	107.926
	REPTree	100.000	91.541

**Table 8:** Recapitulation table to identify the best regression model with respect to the considered evaluation metric.

	CC	MAE	RMSE	RAE	RRSE
Dataset 1	MLP	MLP	MLP	MLP	MLP
	SMOreg	RBD	SMOreg	SMOreg	SMOreg
Dataset 2	MLP	SMOreg	IBK	SMOreg	SMOreg
	SMOreg	KStar	KStar	KStar	KStar

**Table 9:** Accuracy and precision results using twenty nine classification models on both datasets.

Strategy	Classifier	Dataset 1		Dataset 2	
		Accuracy	Precision	Accuracy	Precision
Bayes	BayesNet	71.642	0.742	67.000	0.671
	NaiveBayes	80.597	0.833	59.000	0.590
	NaiveBayesUpdateable	80.597	0.833	59.000	0.590
Functions	Logistic	80.597	0.836	83.000	0.833
	MultilayerPerceptron	91.045	0.914	66.000	0.667
	SimpleLogistic	91.045	0.915	67.000	0.670
	SMO	92.537	0.928	66.300	0.660
Lazy	IBK	94.030	0.940	86.000	0.867
	KStar	86.567	0.874	84.000	0.842
	LWL	68.657	0.667	36.000	0.294
Meta	Bagging	74.627	0.778	65.000	0.615
	ClassificationViaRegression	79.105	0.836	57.000	0.571
	FilteredClassifier	76.119	0.788	65.000	0.579
	LogitBoost	91.045	0.914	82.000	0.800
	MultiClassClassifier	85.075	0.869	69.000	0.667
	RandomCommittee	91.045	0.916	83.000	0.842
	RandomizableFilteredClassifier	85.075	0.853	86.000	0.859
	RandomSubSpace	88.060	0.893	66.000	0.671
	Vote	14.925	0.143	16.000	0.160
Rules	DecisionTable	79.105	0.863	50.000	0.583
	JRip	80.597	0.826	58.000	0.667
	OneR	76.119	0.927	38.000	0.458
	PART	88.060	0.884	66.000	0.700
	ZeroR	14.925	0.143	16.000	0.160
Trees	J48	88.060	0.890	63.000	0.625
	LMT	91.045	0.915	85.000	0.800
	RandomForest	94.030	0.940	84.000	0.833
	RandomTree	80.597	0.819	82.000	0.833
	REPTree	74.627	0.788	60.000	0.571

**Table 10:** Recall and F1-measure results using twenty nine classification models on both datasets.

Strategy	Classifier	Dataset 1		Dataset 2	
		Recall	F1-Measure	Recall	F1-Measure
Bayes	BayesNet	0.716	0.720	0.670	0.667
	NaiveBayes	0.806	0.806	0.590	0.706
	NaiveBayesUpdateable	0.806	0.806	0.590	0.706
Functions	Logistic	0.806	0.804	0.830	0.870
	MultilayerPerceptron	0.910	0.910	0.660	0.737
	SimpleLogistic	0.910	0.910	0.670	0.667
	SMO	0.925	0.925	0.680	0.632
Lazy	IBK	0.940	0.947	0.860	0.876
	KStar	0.866	0.864	0.840	0.869
	LWL	0.687	0.671	0.307	0.296
Meta	Bagging	0.746	0.750	0.650	0.667
	ClassificationViaRegression	0.791	0.796	0.570	0.591
	FilteredClassifier	0.761	0.764	0.650	0.629
	LogitBoost	0.910	0.912	0.820	0.849
	MultiClassClassifier	0.851	0.850	0.688	0.697
	RandomCommittee	0.910	0.911	0.830	0.859
	RandomizableFilteredClassifier	0.851	0.850	0.860	0.870
	RandomSubSpace	0.881	0.881	0.660	0.667
	Vote	0.149	0.144	0.160	0.164
Rules	DecisionTable	0.791	0.809	0.500	0.533
	JRip	0.806	0.806	0.580	0.600
	OneR	0.761	0.805	0.380	0.500
	PART	0.881	0.880	0.700	0.765
	ZeroR	0.149	0.146	0.160	0.216
Trees	J48	0.881	0.881	0.630	0.625
	LMT	0.910	0.910	0.850	0.842
	RandomForest	0.940	0.941	0.840	0.889
	RandomTree	0.806	0.808	0.820	0.859
	REPTree	0.746	0.753	0.600	0.667

**Table 11:** MCC results using twenty nine classification models on both datasets.

Strategy	Classifier	Dataset 1	Dataset 2
		MCC	MCC
Bayes	BayesNet	0.651	0.664
	NaiveBayes	0.763	0.719
	NaiveBayesUpdateable	0.763	0.719
Functions	Logistic	0.766	0.861
	MultilayerPerceptron	0.887	0.721
	SimpleLogistic	0.887	0.700
	SMO	0.904	0.687
Lazy	IBK	0.930	0.862
	KStar	0.831	0.859
	LWL	0.677	0.293
Meta	Bagging	0.686	0.634
	ClassificationViaRegression	0.749	0.634
	FilteredClassifier	0.710	0.645
	LogitBoost	0.887	0.853
	MultiClassClassifier	0.819	0.704
	RandomCommittee	0.888	0.853
	RandomizableFilteredClassifier	0.810	0.862
	RandomSubSpace	0.854	0.693
	Vote	−0.143	0.176
Rules	DecisionTable	0.778	0.595
	JRip	0.757	0.667
	OneR	0.787	0.457
	PART	0.849	0.808
	ZeroR	−0.143	0.256
Trees	J48	0.852	0.692
	LMT	0.887	0.839
	RandomForest	0.926	0.862
	RandomTree	0.757	0.861
	REPTree	0.691	0.612

**Table 12:** Recapitulation table to identify the best classification model.

	Accuracy	Precision	Recall	F1-Measure	MCC
Dataset 1	IBK	IBK	IBK	IBK	IBK
	RF	RF	RF	RF	RF
Dataset 2	IBK	IBK	IBK	RF	IBK
	RFC	RFC	RFC	IBK	RF

**Table 13:** Best learning strategy results.

Task		Bayes	Function	Lazy	Meta	Rules	Trees
Regression	CC		0.851	0.847	0.375	0.191	0.422
	CC		0.641	0.647	0.318	0.206	0.510
	MAE		0.559	0.548	0.744	1.036	0.985
	MAE		2.985	2.797	3.082	3.216	3.303
	RMSE		0.681	0.759	1.163	1.221	1.161
	RMSA		3.851	3.490	4.145	4.156	4.188
	RAE		43.729	42.911	75.026	81.103	77.106
	RAE		79.773	74.762	87.815	85.969	88.289
	RRSE		47.196	52.611	80.590	84.615	80.451
	RRSE		82.007	78.779	87.840	88.508	89.193
Classification	Accuracy	77.612	88.806	83.085	76.120	67.761	85.672
	Accuracy	61.667	70.575	68.667	65.444	45.600	74.800
	Precision	0.803	0.898	0.827	0.777	0.729	0.870
	Precision	0.617	0.708	0.668	0.640	0.514	0.732
	Recall	0.776	0.888	0.831	0.761	0.678	0.857
	Recall	0.617	0.710	0.669	0.654	0.464	0.748
	F1-measure	0.777	0.887	0.827	0.762	0.689	0.859
	F1-measure	0.693	0.727	0.680	0.666	0.523	0.776
	MCC	0.726	0.861	0.843	0.696	0.606	0.823
	MCC	0.701	0.742	0.716	0.673	0.557	0.773

## Data Availability

Data are contained within the article.
